# Patient-Reported Outcomes From Patients With Heart Failure Participating in the Future Patient Telerehabilitation Program: Data From the Intervention Arm of a Randomized Controlled Trial

**DOI:** 10.2196/26544

**Published:** 2021-07-02

**Authors:** Cathrine Skov Schacksen, Anne-Kirstine Dyrvig, Nanna Celina Henneberg, Josefine Dam Gade, Helle Spindler, Jens Refsgaard, Malene Hollingdal, Lars Dittman, Kim Dremstrup, Birthe Dinesen

**Affiliations:** 1 Laboratory for Welfare Technology - Telehealth & Telerehabilitation, Sport Sciences - Performance and Technology, Department of Health Science and Technology Aalborg University Aalborg East Denmark; 2 The Danish Clinical Quality Program– National Clinical Registries (RKKP) Odense Denmark; 3 Department of Psychology and Behavioral Sciences Aarhus University Aarhus Denmark; 4 Cardiology Ward Regional Hospital Viborg Viborg Denmark; 5 Networks Technology and Service Platforms, DTU Fotonik, Department of Photonics Engineering Technical University of Denmark Kgs Lyngby Denmark; 6 Department of Health Science and Technology Aalborg University Aalborg Denmark

**Keywords:** adherence, cardiology, cardiomyopathy, compliance, heart failure, heart, Kansas City Cardiomyopathy Questionnaire, monitoring, patient-reported outcome, patients, quality of life, rehabilitation, self-reporting, telehealth, telemonitoring

## Abstract

**Background:**

More than 37 million people worldwide have been diagnosed with heart failure, which is a growing burden on the health sector. Cardiac rehabilitation aims to improve patients’ recovery, functional capacity, psychosocial well-being, and health-related quality of life. However, cardiac rehabilitation programs have poor compliance and adherence. Telerehabilitation may be a solution to overcome some of these challenges to cardiac rehabilitation by making it more individualized. As part of the Future Patient Telerehabilitation program, a digital toolbox aimed at enabling patients with heart failure to monitor and evaluate their own current status has been developed and tested using data from a patient-reported outcome questionnaire that the patient filled in every alternate week for 1 year.

**Objective:**

The aim of this study is to evaluate the changes in quality of life and well-being among patients with heart failure, who are participants in the Future Patient Telerehabilitation program over the course of 1 year.

**Methods:**

In total, 140 patients were enrolled in the Future Patient Telerehabilitation program and randomized into either the telerehabilitation group (n=70) or the control group (n=70). Of the 70 patients in the telerehabilitation group, 56 (80.0%) answered the patient-reported outcome questionnaire and completed the program, and these 56 patients comprised the study population. The patient-reported outcomes consisted of three components: (1) questions regarding the patients’ sleep patterns assessed using the Spiegel Sleep Questionnaire; (2) measurements of physical limitations, symptoms, self-efficacy, social interaction, and quality of life assessed using the Kansas City Cardiomyopathy Questionnaire in 10 dimensions; and (3) 5 additional questions regarding psychological well-being that were developed by the research group.

**Results:**

The changes in scores during 1 year of the study were examined using 1-sample Wilcoxon signed-rank tests. There were significant differences in the scores for most of the slopes of the scores from the dimensions of the Kansas City Cardiomyopathy Questionnaire (*P*<.05).

**Conclusions:**

There was a significant increase in clinical and social well-being and quality of life during the 1-year period of participating in a telerehabilitation program. These results suggest that patient-reported outcome questionnaires may be used as a tool for patients in a telerehabilitation program that can both monitor and guide patients in mastering their own symptoms.

**Trial Registration:**

ClinicalTrials.gov NCT03388918; https://clinicaltrials.gov/ct2/show/NCT03388918

## Introduction

Cardiovascular diseases are the leading cause of death worldwide [1]. In 2016, cardiovascular diseases were the cause of 31% of all deaths, which corresponded to 17.9 million people [1]. More than 37 million people worldwide have been diagnosed with heart failure (HF). Because of a poor prognosis, a high risk of disadvantageous outcomes, and increasing prevalence, HF is a growing burden on the health sector [2-4]. Cardiac rehabilitation aims to improve patients’ recovery, functional capacity, psychosocial well-being, and health-related quality of life. The rehabilitation process combines activities such as physical activity, improved diet, weight control, psychosocial coping, and disease management [5]. However, cardiac rehabilitation programs have poor compliance and adherence. Patients may have poor means of transport to the rehabilitation facility, lack motivation, and feel that rehabilitation activities are not sufficiently individualized; all of these barriers negatively impact adherence to rehabilitation programs, which may in turn exacerbate symptoms including edema, fatigue, and shortness of breath, thus leading to readmissions [5,6]. Telerehabilitation (TR) may be a solution to overcome some of these challenges to cardiac rehabilitation [7,8]. TR is defined as the delivery of rehabilitation services through information and communication technologies [9].

TR may also be clinically relevant in obtaining health status measures from the patients. In turn, these measures add information regarding the severity of HF and may be used as an aid for clinical management [10]. Patient-reported outcomes (PROs) are clinical outcomes that increasingly focus on reducing the disease burden and improving general well-being and lifespan [11]. PRO can be used as a tool for screening and monitoring symptoms and assessing the course of the disease over time for clinicians to evaluate patient symptoms [12]. In a PRO regime, the outcome of a treatment is directly self-reported by the patient without registration or interpretation by a clinician [11]. Some of the outcomes are measurements of the patient’s symptoms and health-related quality of life, which enable PROs to enhance targeted care and contribute to the optimal use of health care resources [11]. In this study, PRO from the Future Patient Telerehabilitation (FPT) program will be made available to patients as a tool for empowering them and increasing their knowledge of their own disease.

Through a user-driven innovation process, we have developed the FPT program for patients with HF. The overall purpose of the FPT program has been to increase the quality of life for patients with HF and to educate the patients to perform individualized monitoring to detect worsening of their own symptoms, thereby avoiding rehospitalization [13]. As part of the FPT program, a digital toolbox containing a PRO questionnaire was created. The purpose of the digital toolbox was to enable HF patients to monitor and evaluate their own current status over the 1-year duration of the TR program, thus enabling them to facilitate their contact with the hospital or their consulting general practitioners. To our knowledge, no previous studies that have investigated the clinical and psychological value of PROs in TR for patients with HF. A review from 2016 [14] on the use of PRO instruments in HF management concluded that the Minnesota Living with Heart Failure and Kansas City Cardiomyopathy Questionnaire (KCCQ) were useful PRO instruments in clinical care. However, more studies are needed on the value and interpretability of PRO instruments in clinical settings. The aim of this study is to evaluate the changes in quality of life and well-being among patients with HF, who are participants in the FPT program over the course of 1 year [13].

## Methods

### Ethical Considerations

This study utilized data from an intervention group that received TR (the TR group) in the FPT study, which was approved by the North Denmark Region Committee on Health Research Ethics (N-20160055) and the Danish Data Protection Agency. The study is registered on ClinicalTrials.gov (NCT03388918). The study was conducted in accordance with the tenets of the Helsinki declaration, and all participants signed an informed consent form prior to enrollment in the study.

### Context and Intervention of the Study

**Figure 1:**
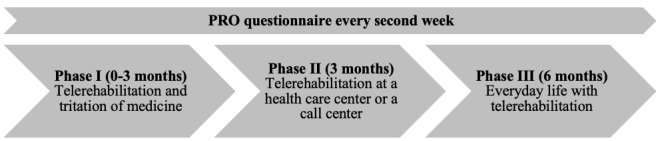
The 3 phases of the Future Patient Telerehabilitation study. PRO: patient-reported outcome.

**Figure 2:**
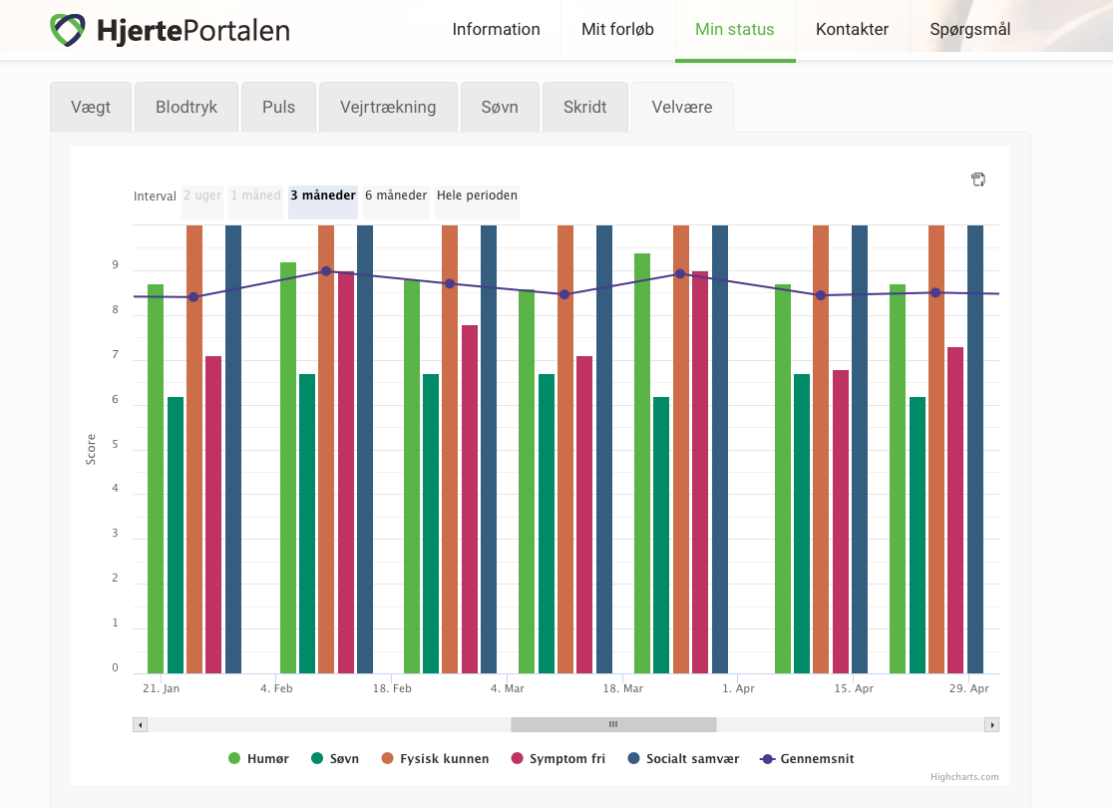
A screen capture of HeartPortal. An illustration of the patient-reported outcome. Row 1: Information, My Treatment, My Status, Contacts, and Questions; Row 2: Weight, Blood Pressure, Pulse, Breathing, Sleep, Steps, and Well-being; Row 3: Time Intervals (3 months, 6 months, and Entire period); and Row 4 (bottom): Mood (light-green dot), Sleep (dark-green dot), Physical condition (orange dot), Symptom-free (red dot), Social contact (blue dot), and Mean (blue line).

The overall aim of the FPT study was to increase the quality of life of patients with HF by training them to perform individualized monitoring, which would enable to detect worsening of their symptoms in a timely manner, thereby avoiding rehospitalization [13]. The intervention of the FPT was divided into three phases (Figure 1): (1) TR and titration of medicine; as the adjustment of medication is specific to each patient, this phase will last 0-3 months; (2) TR at home and at a health care center or call center (3 months); and (3) follow-up with TR in everyday life (6 months). The TR program was based on a webpage called the HeartPortal [15], which is a digital toolbox that functions as an interactive learning module. The HeartPortal consists of (1) an information page containing text and short videos, (2) a communication platform that helps patients design their own TR plan and communicate directly with health care professionals, (3) visualization of measured values, and (4) a PRO questionnaire to be answered every second week. The measured values in HeartPortal included the patients’ vital signs such as blood pressure, daytime and nighttime pulse rates, weight, step count, respiration, and hours of sleep. All data measured from the technologies were transmitted by the patient to HeartPortal. The data are illustrated as graphs and can be visualized and shared among patients, their relatives, and health care professionals. Upon enrollment in the study, the patients were instructed on how to use the PRO data to monitor their own disease and how to take necessary action if their symptoms worsened. The patients had the opportunity to contact the TR coordinator of the FPT program regarding any necessary action to be taken. Figure 2 shows the patients’ PRO data in graphical format over a period of 2 months. The control group participated in the same 3 phases but without participating in the TR program; that is, they had no access to HeartPortal.

### Participants

Participants were recruited from the cardiology wards at hospitals in Skive, Viborg, Silkeborg, and Randers in Denmark. Participants were recruited by a project nurse. The inclusion criteria for the FPT were the following: patients with HF with a New York Heart Association (NYHA) functional class of I-IV, of whom a maximum of 20% of the patients were of NYHA class I, ≥18 years of age, able to care for themselves, and had basic computer skills.

### Sample Size

The sample size of the FPT study was determined to be 70 patients in each group (assuming a normal distribution), a power of 80%, and a potential 10% dropout. This calculation was based on the KCCQ guidelines, which state that a “moderate” level of improvement is equal to a 10-point increase in the KCCQ score [13,16]. In this study of the FPT program, only the KCCQ outcomes from the intervention are reported. A comparison of the KCCQ results from both the intervention and control groups will be reported in a subsequent study on the evaluation of health utilizations.

### One Arm of a Randomized Controlled Trial

**Figure 3:**
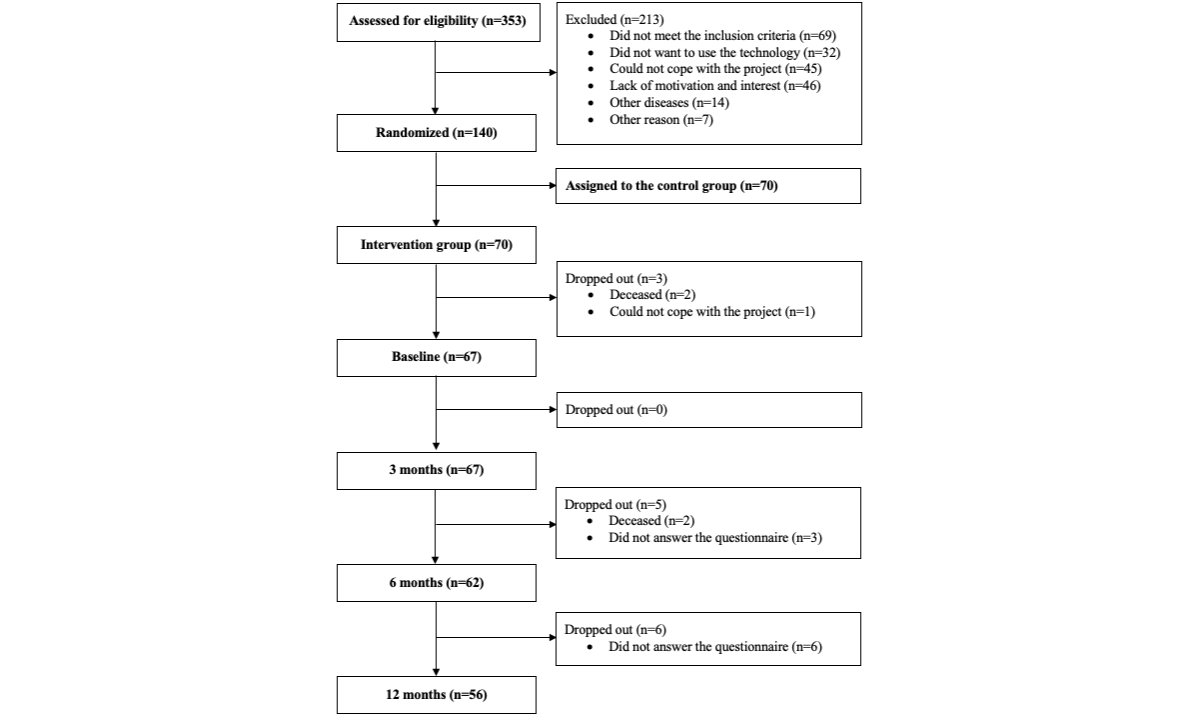
CONSORT (Consolidated Standards of Reporting Trials) diagram for the intervention group of the Future Patient Telerehabilitation trial.

In total, 140 patients were enrolled in the FPT and randomized into either the TR group (n=70) or the control group (n=70) [13]. This study only reports the findings of the TR group. Of the 70 patients in the TR group, 56 (80.0%) answered the PRO questionnaire and completed the program, and these 56 patients constituted the study population. The randomization and follow-up procedure for the patients in the TR group are shown in the CONSORT (Consolidated Standards of Reporting Trials) diagram in Figure 3.

### Sociodemographic and Clinical Data

Sociodemographic data (age, gender, education, employment status, and civil status) and clinical data (etiology of heart failure, NYHA class, ejection fraction, weight, blood pressure, and heart rate) were collected through self-reports or from the electronic patient record.

### PRO Measures

The PRO questionnaire consisted of components from three questionnaires: (1) patients’ sleep quality was evaluated using the Spiegel Sleep Questionnaire [17]; (2) physical limitations, symptoms, self-efficacy, social interactions, and quality of life were assessed using the validated KCCQ [16,18]; and (3) psychological well-being was evaluated using 5 additional questions developed by the research group.

#### Spiegel Sleep Questionnaire

Sleep quality was measured using the Spiegel Sleep Questionnaire [17]. The questionnaire consists of 6 questions regarding the patients’ sleep patterns and sleep quality, with all items scored using a 5-point Likert scale. This is a validated sleep questionnaire and has been used in clinical studies [17,19].

#### KCCQ

Measures of physical limitations, symptoms, self-efficacy, social interaction, and quality of life were self-assessed using the validated KCCQ. The KCCQ is a 23-item self-administered questionnaire with 15 questions. All items are scored on a 5-7–point Likert scale. There are 5 individual subscales, all of which, except for the self-efficacy subscale, are aggregated into the clinical and overall summary scores. The total score of the questionnaire is calculated by assigning an ordinal value to each response, with 1 as the lowest value, and then adding the values to obtain a scaled score for each domain. Accordingly, the scaled scores range from 0 to 100, with a higher score indicating a better health status, fewer symptoms, and increased quality of life. Missing responses are assigned a value that corresponds to an average of the answered items within the domain [16,20].

#### Psychological Well-being

The psychological well-being of the participants was measured using 5 questions developed by the research group. The questions were validated for clarity and understanding among patients with HF before use, in an iterative process. The questions were answered using a visual analogue scale, with 0 being the lowest value and 9 being the highest value. The 5-item psychological well-being scale was constructed on the basis of 5 different psychological aspects known to be of importance in HF (depression, anxiety, positive affect, hopelessness, and social support). We chose this approach, as it was not feasible to measure these factors using 5 psychological questionnaires in their entirety to measure these aspects. In addition, it has previously been shown that it may be possible to assess some of these factors through very brief questionnaires [21]. Furthermore, these questions were not intended as a means of diagnosis, but rather as indications of the patients’ psychological status at the time of measurement.

### Data Collection

All PRO questionnaire data were collected using Research Electronic Data Capture platform (Vanderbilt University). The questionnaires were made available on the internet to the patients on HeartPortal twice a month (between days 10-14 and 24-28 of the month). If the patients did not answer the questionnaire, the TR coordinator sent them a reminder.

### Data Preprocessing

Data quality was ensured through data preprocessing. The time points for the data were converted from dates to a numeric variable—the questionnaire number. The PRO questionnaires were available to the patients twice a month at the aforementioned timepoints. Consequently, the questionnaires were still available for responses during the entire period and were not withdrawn after being completed by the patient. To correct for multiple responses to the same timepoint, the first responses within each time period were used in further analysis.

### Statistical Analysis

Missing data were imputed by matching the responses from the questionnaires answered to those closest to the timepoint of the missing value [22]. Furthermore, our analyses showed that the imputation strategy did not significantly alter the results (these analyses are not included in this study). Nevertheless, missing data constitute a noteworthy problem. Furthermore, to account for missing data and varying durations of the 3 phases for the individual subjects in the study, the differences in scores in the 3 phases have been compared with trends for the subjects individually, in terms of slopes from linear regression analysis. In addition, when calculating the results for each dimension of the questionnaire, a minimum of half of the questions in each dimension was required. If less than half of the questions were answered, the results from that particular dimension would be excluded from the analysis [22].

All preprocessing steps and data analysis were performed using MATLAB (version R2019a, The MathWorks Inc). All statistical analyses were performed using IBM SPSS Statistics (version 26, IBM Corp).

Prior to analysis, the data were examined for normality of their distribution, using a Shapiro–Wilk test and by visual inspection of scatter plots. The 3 different questionnaires, as well as the subscales, comprising the PRO questionnaire in the FPT program were analyzed individually. To enable comparisons across subscales, scores were standardized by transforming each subscale to a range of 0-100 with higher scores indicating better health.

To evaluate changes in PROs during the 1-year duration of the intervention, Friedman tests were used. In case of significance, Wilcoxon sign-rank post hoc tests were used to determine in which phase the differences occurred during the 1-year duration.

## Results

### Patient Characteristics

Table 1 shows the baseline sociodemographic and clinical characteristics of patients in the TR group. These characteristics are depicted as either the number of patients or as mean (SD) values and ranges for the different parameters.

**Table 1:** Clinical and sociodemographic data of the patients enrolled in the intervention group of the Future Patient Telerehabilitation program (N=67).

Variables				Values
**Age (years), mean (SD); range**				
		Men (n=51)		62.18 (10.64); 35-81
		Women (n=16)		60.31 (11.31); 43-81
		Men and women (n=67)		61.73 (10.75); 35-81
**Clinical parameters, mean (SD); range**				
		Weight (kg)		85.34 (20.35); 56-166
		Systolic blood pressure (mmHg)		124.42 (17.67); 84-172
		Diastolic blood pressure (mmHg)		78.97 (10.99); 48-122
		Heart rate (beats/minute)		78.70 (17.76); 46-119
		Ejection fraction (%)		31.80 (8.49); 10-45
**Number of patients by New York Heart Association class, n (%)**				
	I		10 (14.9)	
	II		42 (62.7)	
	III		13 (19.4)	
	IV		2 (2.9)	
**Number of patients by the etiology^a^ of heart failure, n (%)**				
		Ischemia		32 (47.8)
		Idiopathy		17 (25.4)
		Hypertension		6 (8.9)
		Valvular heart disease		8 (11.4)
		Alcoholism		0 (0.0)
		Postpartum heart failure		0 (0.0)
		Chemotherapy		0 (0.0)
		Others		18 (26.9)
**Marital status, n (%)**				
		Single or living alone		24 (35.8)
		Married or living with a partner		43 (64.2)
**Education level, n (%)**				
		Primary school		4 (5.9)
		Unskilled		16 (23.9)
		Skilled worker		30 (44.8)
		High school		5 (7.5)
		Bachelor’s degree		9 (13.4)
		Master’s degree		2 (2.9)
		Doctoral degree		1 (0.7)
**Employment status, n (%)**				
		Unemployed		0 (0.0)
		Sick leave		19 (28.4)
		Working for <20 hours/week		5 (7.5)
		Working for 20-36 hours/week		2 (2.9)
		Working full-time for 37 h/week		9 (13.4)
		Retired		32 (47.8)

^a^Some patients have more causes of etiology of heart failure.

### Well-being in the 3 Phases of the Study

The intervention in the FPT program was divided into 3 phases. The mean participation times for the TR patients in each phase were as follows: (1) TR and titration of medicine (2.37 months, SD 1.72 months), (2) TR at home and at a health care center or call center (3.43 months, SD 0.89 months), and (3) follow-up with TR in everyday life (5.77 months, SD 1.00 month). The patients completed 74.93% (SD 23.31%) of the total number of questionnaires, with a minimum compliance of 14.81% and a maximum compliance of 100%.

The Shapiro–Wilk test revealed that the data in the 13 different dimensions of the questionnaire were not normally distributed. Therefore, descriptive statistics for the data in 13 dimensions are presented in Table 2 as median (IQR) scores.

**Figure 4:**
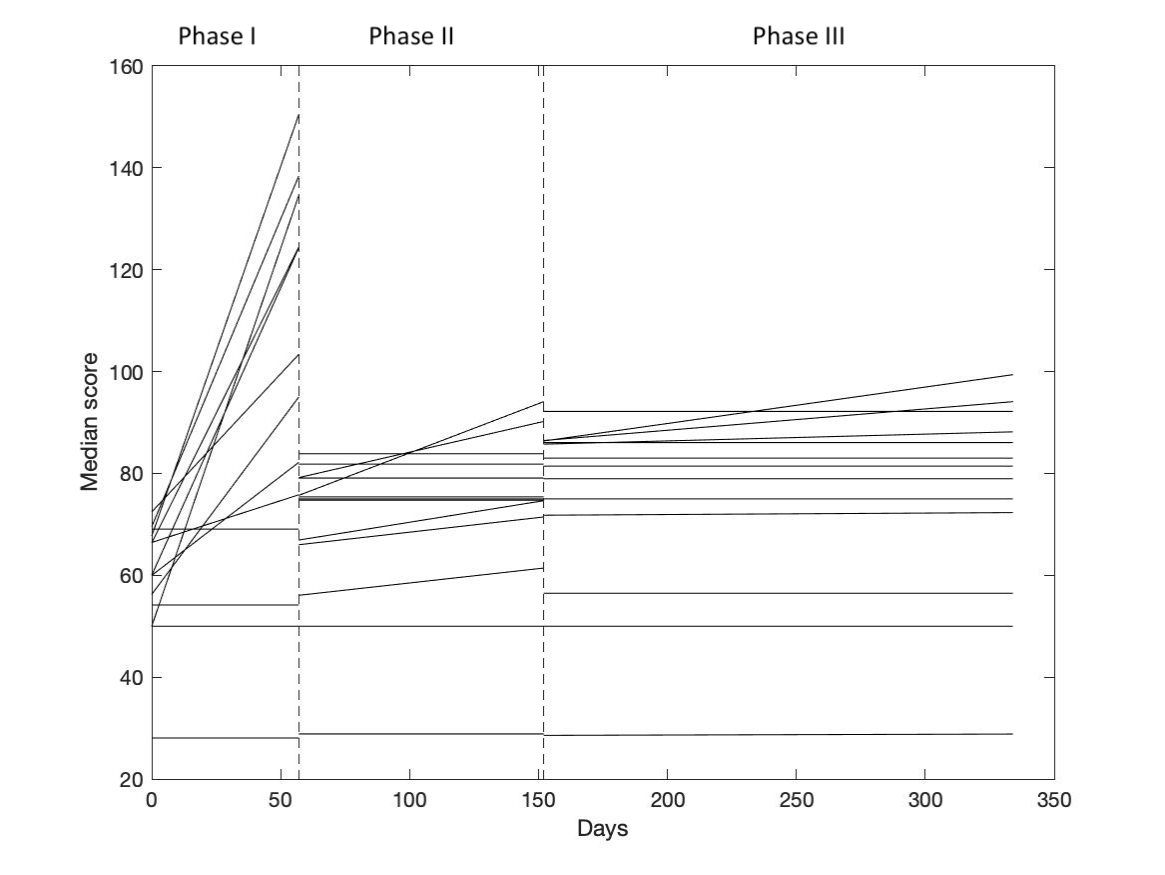
Changes in median scores from the 13 dimensions of the questionnaires. Dotted lines indicate a change in phase in the Future Patient Telerehabilitation program.

Changes in the median scores from each dimension for the 3 phases are illustrated in Figure 4. The dotted lines in Figure 4 demarcate the 3 phases. Each line in Figure 4, within each phase, represents 1 of the 13 dimensions of the questionnaires. As such, Figure 4 illustrates the trend within each of the 3 phases and serves as a visual presentation of the data, showing that all 3 phases have an increasing slope. Based on the changes in the median scores shown in Figure 4, we observed a trend that indicates that the scores increased for most of the dimensions during the 3 phases, most notably in phase 1.

Changes in PRO scores across the 3 phases of the study were examined using Friedman tests. As shown in Table 3, there were significant differences in scores on most of the dimensions in the KCCQ (*P*<.05) during the 1-year intervention. Wilcoxon signed-rank post hoc tests were performed to examine the differences identified by the Friedman tests. These results are presented in Table 4 as *z* scores, which are standardized scores that indicate the difference between preintervention and postintervention scores of the measure in question. As such, a negative *z* score indicates a positive change over time (median scores for each phase are provided in Table 2). However, since no significant differences were observed across phases 2 and 3, these results are not shown.

**Table 2:** Median (IQR) scores for all patient-reported outcome measures.

Questionnaire	Dimension	Median (IQR) score in phase 1 (n=67)	Median (IQR) score in phase 2 (n=62)	Median (IQR) score in phase 3 (n=56)	Median (IQR) score in all phases (n=56)
Spiegel Sleep Questionnaire	Sleep	58.33 (12.50)	58.33 (12.50)	57.20 (12.50)	58.33 (12.50)
Psychological well-being	Psychological well-being	28.89 (8.89)	28.89 (6.67)	28.89 (6.67)	28.89 (5.28)
**Kansas City Cardiomyopathy Questionnaire**					
	Physical limitations	79.17 (31.25)	87.50 (26.56)	91.67 (29.17)	88.75 (29.17)
	Symptom stability	50.00 (0.00)	50.00 (0.00)	50.00 (0.00)	50.00 (0.00)
	Symptom frequency	79.17 (37.50)	77.60 (35.94)	83.33 (37.76)	82.81 (36.98)
	Symptom burden	75.00 (3.50)	75.00 (25.00)	83.33 (35.42)	83.33 (31.25)
	Total symptom score	76.04 (34.37)	78.39 (30.99)	83.33 (34.90)	82.81 (33.20)
	Self-efficacy	75.00 (25.00)	75.00 (25.00)	75.00 (25.00)	75.00 (25.00)
	Quality of life	66.67 (3.33)	75.00 (35.42)	83.33 (32.29)	83.33 (33.33)
	Social limitation	66.67 (46.88)	80.21 (32.29)	83.85 (33.33)	81.25 (37.50)
	Overall summary score	72.14 (32.42)	77.34 (33.28)	82.58 (31.48)	79.75 (30.21)
	Clinical summary score	76.04 (27.08)	79.82 (24.90)	86.98 (32.03)	85.02 (30.14)

**Table 3:** Results of the Friedman test of the individual dimensions during 1 year.

Questionnaire	Dimension	Changes in score over time	
		*χ*^2^ (*df*)	*P* value
Spiegel Sleep Questionnaire	Sleep quality	0.14 (2)	.93
Psychological well-being	Psychological well-being	0.04 (2)	.98
**Kansas City Cardiomyopathy Questionnaire**			
	Physical limitations	9.25 (2)	.01^a^
	Symptom stability	0.75 (2)	.69
	Symptom frequency	16.75 (2)	<.001^a^
	Symptom burden	11.61 (2)	.001^a^
	Total symptom score	17.18 (2)	<.001^a^
	Self-efficacy	4.32 (2)	.12
	Quality of life	7.54 (2)	.02^a^
	Social limitation	19.75 (2)	<.001^a^
	Overall summary score	14.71 (2)	.001^a^
	Clinical summary score	19.54 (2)	<.001^a^

^a^Statistically significant at *P*<.05 (2-tailed).

**Table 4:** *z* scores and significance levels from the Wilcoxon signed-rank post hoc tests when testing for differences in trends on the Kansas City Cardiomyopathy Questionnaire in terms of slopes between the phases.

Dimension	Slopes			
	Phase I vs phase II		Phase I vs phase III	
	*z* score	*P* value	*z* score	*P* value
Physical limitations	–2.41	.02^a,b^	–2.62	.009^a,b^
Symptom frequency	–3.30	.001^a,b^	–3.58	<.001^a,b^
Symptom burden	–1.74	.08^a^	–2.73	.006^a,b^
Total symptom score	–2.77	.006^a,b^	–3.30	.001^a,b^
Quality of life	–1.69	.09^a^	–3.15	.002^a,b^
Social limitation	–2.85	.004^a,b^	–3.33	.001^a,b^
Overall summary score	–2.27	.006^a,b^	–3.83	<.001^a,b^
Clinical summary score	–3.76	<.001^a,b^	–3.90	<.001^a,b^

^a^Statistically significant at *P*<.05.

^b^Higher slopes in phase I.

## Discussion

### Principal Findings

In the FPT program, the PRO questionnaire has served as a tool for patients on HeartPortal to help themselves monitor their well-being. The aim of this study was to evaluate the changes in quality of life and well-being for patients with HF who are participants in the FPT program over a 1-year period. We found that during the 1-year intervention, the following dimensions showed an increase in their median scores: physical limitation, symptom frequency, total symptom score, quality of life, social limitation, overall summary score, and clinical summary score. These changes were significantly different for most of the change in scores over time from the dimensions from the KCCQ.

In Figure 4, the increase in scores appeared more pronounced in the first phase, where patients start their TR and have their medication adjusted, compared to phases II (TR at home and rehabilitation at a health care center) and III (TR at home and follow-up in everyday life). However, patient scores increased continuously throughout all phases. This suggests that the intervention is most effective in phase I, as patients in this initial phase will tend to be more open-minded and motivated for changing their lifestyle and using the digital toolbox to empower themselves. As such, our results support the notion that rehabilitation should be initiated as soon as possible, preferably as part of the initial treatment phase, when patients are most motivated to initiate such changes. An analysis of the changes in scores during a year of TR showed significant differences in the scores on all dimensions of the KCCQ, except for self-efficacy. In general, these findings indicate that almost all scores from the different dimensions showed an increase and a significant difference for the overall change during 1 year of the intervention, thus indicating an improvement in the patients’ health. We have not identified other studies reporting this type of improvement by using PRO questionnaires.

PRO questionnaires are normally used as a tool for research. They enable clinicians to obtain a better understanding of the patients’ health status and serve as a clinical management tool [10]. In the FPT program, we deployed PRO as a tool for patients to monitor their own disease during their rehabilitation process. The patients answered almost 75% of all questionnaires for a period of 1 year, thus indicating a high degree of compliance with the PRO tool on HeartPortal. A study in Denmark [23] on HF and PRO has reported a compliance rate of approximately 50%. In this study, however, the PRO questionnaire was used by patients to document their symptoms prior to visiting the HF outpatient clinic at the hospital. Thus, PRO served as a tool for clinicians as well [23]. This active use of PRO data by patients may help explain the high compliance rate in our FPT program. To our knowledge, no other TR studies have allowed for the possibility of evaluating the current status of patients with HF during 1 year with the use of PRO measures. Our data analysis has thus demonstrated that the PRO questionnaire can provide a cross-sectional view of the development of the patients’ well-being and quality of life. The increase in the scores over time may indicate that the patients have used the PRO questionnaire to become more aware of their own symptoms and, therefore, be better equipped to navigate and cope with HF in their everyday lives. We have explored how patients have used the PRO questionnaire in the digital toolbox during their participation in the FPT program. This will be further documented in a subsequent study that describes patients’ qualitative perspectives of using PRO as a part of TR.

A new study by Butler et al [24] in 2020 suggests that changes even smaller than 5-point improvements in KCCQ scores may be clinically significant. In the FPT study, the median (IQR) of the KCCQ clinical summary score increased from 76.04 (IQR 27.08) in phase I to 86.98 (IQR 32.03) in phase III, yielding a total median increase of more than 10 points. This indicates that the change in scores has clinical relevance, thereby indicating improvements in health based on the KCCQ results. However, no change was observed in the median scores of the Spiegel Sleep Questionnaire or the psychological well-being questionnaire. However, the FPT program was not designed to provide a specialized psychological intervention for psychological distress, such as anxiety and depression, but followed general guidelines for identifying and treating psychological distress in patients with HF.

### Limitations

This study has limitations that should be considered. First, the timing of the PRO questionnaires may have been too frequent. In this study, it was collected every second week during 1 year, and this may have resulted in some patients skipping some of the questionnaires, thereby resulting in missing data. However, as some of the questionnaires referred to the patients’ perceived symptoms over the previous 2 weeks, we considered this a relevant timeframe to detect changes in symptoms. Moreover, the responses from the PRO questionnaire provide subjective cross-sectional insights into the patients’ well-being, which should be taken into consideration when evaluating their general well-being and when used in a clinical setting. In future studies, technological opportunities for mandatory responses may be used to generate more complete data from all participants.

It would have been valuable to include data from the control group for comparison. This study compared individual data over time, which is a valuable approach in identifying a trend. Nevertheless, on the basis of the available data, it was not possible to assess the development in quality of life and clinical aspects within the control group.

### Conclusions

There was a significant increase in clinical and social well-being and quality of life during 1 year of participating in the TR program. These results suggest that PRO questionnaires may be used as a tool for patients in a TR program that can both monitor and guide the patients in mastering their own symptoms, improving their own well-being in a TR program, and enhancing their recovery.

## Abbreviations

FPT: Future Patient Telerehabilitation
HF: heart failure
KCCQ: Kansas City Cardiomyopathy Questionnaire
NYHA: New York Heart Association
PRO: patient-reported outcome
TR: Telerehabilitation
